# A case of acute coronary syndrome secondary to cocaine-induced coronary thrombosis

**DOI:** 10.1093/ehjcr/ytaf303

**Published:** 2025-06-23

**Authors:** Mahmoud Morsy, Saad Fyyaz, Magda-Madalina Olaru, Maciej Marciniak

**Affiliations:** Cardiology Department, Atkinson Morley Wing, St George’s University Hospital, NHS foundation Trust, London SW17 0QT, United Kingdom; Cardiology Department, Atkinson Morley Wing, St George’s University Hospital, NHS foundation Trust, London SW17 0QT, United Kingdom; Cardiology Department, Atkinson Morley Wing, St George’s University Hospital, NHS foundation Trust, London SW17 0QT, United Kingdom; Cardiology Department, Atkinson Morley Wing, St George’s University Hospital, NHS foundation Trust, London SW17 0QT, United Kingdom

## Case description

**Figure 1:**
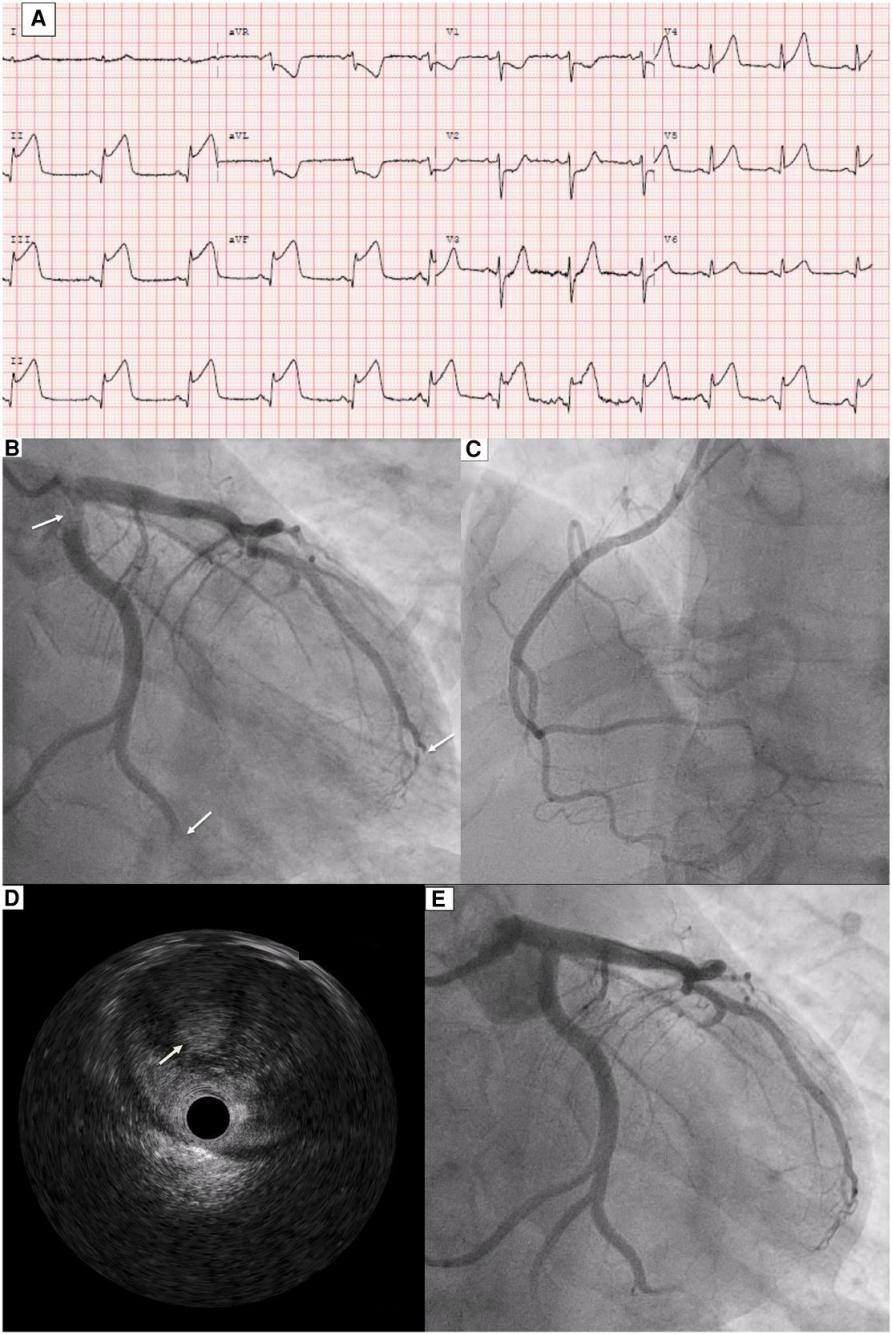
Electrocardiogram at presentation showing evidence of inferior ST elevation myocardial infarction (*A*). Coronary angiogram (dominant left system) showing evidence of thrombosis in the ostial, proximal, and the distal segments of the left circumflex artery and in distal segment of the left anterior descending artery (arrows) (*B*). No evidence of thrombosis in the non-dominant right coronary artery (*C*). Intravascular ultrasound showed heavy thrombosis with otherwise healthy underlying vessels (*D*). Follow-up coronary angiogram after 3 days showing resolution of coronary thrombosis with improved flow in the coronaries (*E*).

A 39-year-old gentleman with no significant past medical history apart from smoking and cocaine abuse presented to our hospital with central chest pain and diaphoresis, which started 12 h before admission. An electrocardiogram showed inferior ST elevation (*Figure 1A*). He was transferred immediately to the Cath lab, where he underwent a coronary angiogram that showed thrombus in the ostial to proximal segment of the left circumflex (LCX) artery, with thrombus in the distal LCX, and thrombus in the distal segment of the left anterior descending artery (*Figure 1B*; Supplementary material online, *Video S1*) and a normal right coronary artery (*Figure 1C*). Intravascular ultrasound showed heavy thrombosis with otherwise healthy underlying vessels (*Figure 1D*). Thrombus aspiration was not successful due to late presentation. Given the heavy thrombosis in the ostial LCX and potential compromise to the left main artery, a multidisciplinary team discussion was held, and a decision was made to proceed with thrombolysis using Tenecteplase 40 mg, followed by anticoagulation with low molecular weight heparin and a follow-up coronary angiogram in 48–72 h. Vasculitis and prothrombotic screens were negative. A bedside transthoracic echocardiogram showed mildly impaired left ventricular systolic function, with an ejection fraction of 45%, with hypokinetic mid to apical inferior and inferoseptal segments (see Supplementary material online, *Videos S3* and *S4*). A bubble contrast study showed no evidence of right-to-left shunt (see Supplementary material online, *Video S5*). Follow-up coronary angiogram showed resolution of coronary thrombosis with improvement of coronary flow (*Figure 1E*; Supplementary material online, *Video S2*). The patient was discharged on apixaban and clopidogrel for 3 months, followed by aspirin for life.

Cocaine use has been reported in the literature to cause coronary thrombosis by inducing platelet aggregation, even in normal coronaries.^1^ Myocardial infarction was reported in 6% of patients presenting with chest pain after cocaine use in two different studies.^2^ In a study performed on 71 cocaine abusers, there was a significant increase in the risk of stent thrombosis in cocaine abusers compared with the control group; thus, effort should be made to avoid stents where possible.^3^ Fibrinolysis was reported to be safe in patients with myocardial infarction secondary to cocaine abuse.^4^ For secondary prevention, cessation of cocaine use, modification of atherosclerotic risk factors, and the use of aspirin, clopidogrel, and direct thrombin inhibitors have been reported in the literature.^2^

## Supplementary Material

ytaf303_Supplementary_Data

## Data Availability

Data is available upon request from the corresponding author.

## Notes

**Consent:** The authors confirm that written consent for submission and publication of this manuscript including the images and associated text has been obtained from the patient in line with the COPE guidelines.

**Funding:** None declared.
